# Probing the anharmonicity of vibrational polaritons with double-quantum two-dimensional infrared spectroscopy

**DOI:** 10.1515/nanoph-2023-0683

**Published:** 2024-01-10

**Authors:** Shmuel Sufrin, Bar Cohn, Lev Chuntonov

**Affiliations:** Schulich Faculty of Chemistry, Solid State Institute, and Helen Diller Quantum Center, Technion – Israel Institute of Technology, Haifa 3200003, Israel

**Keywords:** vibrational polaritons, anharmonicity, double-quantum 2DIR spectroscopy, infrared surface lattice resonances

## Abstract

Strong coupling between the molecular vibrations and electromagnetic fields of light confined to an infrared cavity leads to the formation of vibro-polaritons – quasi-particles thought to provide the means to control the rates of chemical reactions inside a dark cavity. Despite the mechanisms indicating how vibrational coupling to the vacuum fields can affect the reaction rates are still not well understood, it has been recently demonstrated that the formation of the polariton states alters the ultrafast relaxation dynamics of the strongly coupled system. The relaxation dynamics in molecules, which is known to be important for the chemical reactivity, is directed by anharmonic couplings involving multiple intra- and inter-molecular vibrational degrees of freedom. However, the impact of the molecular anharmonicity on the polariton states remains elusive. Some theoretical models, employed to interpret the experimental observations, assume that vibrational polaritons are harmonic. Others assume a certain anharmonicity of vibro-polaritons; however, to date, it has not been experimentally determined. Herein, we performed double-quantum two-dimensional third-order nonlinear infrared spectroscopy of the carbonyl stretching (C=O) vibrational modes in a thin film of polymethyl methacrylate polymer (PMMA) strongly coupled to the surface lattice resonances of the periodic arrays of half-wavelength infrared disk antennas. We found that, indeed, the mechanical anharmonicity of polaritons is very small. Quantitatively, our results place an upper bound on a polariton mechanical anharmonicity of 2 cm^−1^, compared with that of the C=O mode in a PMMA film of 15 cm^−1^. Thus, our results support previous assumptions regarding the harmonic character of vibro-polaritons.

## 1 Introduction

Vibrational polaritons are quasiparticles that emerge upon strong coupling between infrared-active molecular vibrations and resonant photonic modes of an infrared cavity [1], [2]. Especially great interest in vibrational polaritons arose recently because they are extensively explored in the emerging field of polariton chemistry, where chemical transformations are thought to be controlled by the formation of these hybrid light–matter quantum states [3], [4], [5]. Each molecule in the ensemble subjected to the electric field of the infrared photonic mode simultaneously participates in two collective quasiparticle states known as the lower (LP) and upper (UP) polaritons, and in the manifold of the reservoir (*R*) states. Typically, polaritons have comparable percentages of photonic and molecular components, whereas reservoir states are predominantly molecular [6]. Since the number of molecules needed to realize a vibrational strong coupling regime is generally very large, *N* ∼ 10^9^, the Hopfield coefficients that describe the contribution of each individual molecule to the polariton states are very small. Nevertheless, it is proposed that such collective coupling between the molecules and light can lead to changes in various aspects of molecular behavior, which are manifested on a macroscopic scale [7].

The mechanisms by which the coupling to the cavity can possibly affect the chemical reactions are far from being well understood [8]. One of the interesting directions being investigated concerns the pathways of the intramolecular vibrational energy redistribution (IVR), which can be altered by vibrational strong coupling [9], [10]. It is widely recognized that IVR, which is directed by the anharmonic couplings between different molecular vibrational modes [11], can play an important role in the reaction dynamics [12]. The anharmonic couplings in molecules are manifested by the deviations of the transition frequencies of the high-order vibrational excitations from the corresponding fundamental transition frequencies, as well as in the ultrafast dynamics of the vibrational excitation transfer and relaxation [13].

Femtosecond third-order nonlinear infrared spectroscopy is a powerful method widely employed to study ultrafast molecular vibrational dynamics [14]. In these experiments, the molecular ensemble is illuminated with sequences of three laser pulses, which excite transitions between the ground state $\left|0\right\rangle$ and the first excited state $\left|i\right\rangle$ of the resonant molecular vibrational mode *i* at the fundamental frequency *ω*_0,*i*_, as well as transitions between $\left|i\right\rangle$ and the second excited state $\left|2i\right\rangle$ at the frequency *ω*_*i*,2*i*_ = *ω*_*i*_ − Δ_*i*_, where Δ_*i*_ is the mode’s anharmonicity constant. The third-order nonlinear signals, observed at the *ω*_0,*i*_ transition frequency, correspond to the photoinduced bleach of the ground state population (GSB) and to the stimulated emission (SE) from the first excited state to the ground state, whereas those observed at *ω*_*i*,2*i*_ correspond to the absorption from the first excited state to the second excited state (ESA). In the harmonic system Δ_*i*_ = 0, and since the GSB/SE and ESA signals at *ω*_0,*i*_ and *ω*_*i*,2*i*_ have similar magnitudes but appear out of phase and interfere destructively, such systems cannot be spectroscopically observed by a third-order spectroscopy. The deviation from the harmonic behavior is frequently classified as the mechanical anharmonicity, which appears because of the shape of the molecular potential, or as electrical anharmonicity, which appears because of the nonlinear dependence of the transition dipole moment on the normal coordinates. Thus, even a small anharmonicity (either mechanical or electrical) is sufficient and most high-frequency molecular vibrational modes can be readily detected when interrogated by the nonlinear infrared spectroscopy [15].

Ultrafast infrared spectroscopy is also very useful for studying vibrational polaritons [16], [17], [18]. For example, in the two-dimensional realization of a third-order nonlinear spectroscopy experiment, 2DIR, the spectral data are spread out in two dimensions, revealing the correlations between the excitation and the detection frequencies of the signal. Recently, 2DIR studies identified the flow of the excitation energy along the transfer pathways within vibrational strongly coupled systems, which are established by the synchronized collective motions of the large number of molecules [17], [19], [20]. In contrast to molecules, where the relaxation proceeds via the IVR pathways, the relaxation of polaritons involves equilibration of the vibrational energy excess between the polariton states as well as the transfer into the reservoir states of the strongly coupled system. Such newly formed channels of energy redistribution, which are not present in molecules outside the cavity, can be important not only for the ultrafast dynamics of vibrational excitations hybridized with photonic modes, but also for the chemical reactivity of the strongly coupled molecules [21], [22].

Interpreting the spectroscopic observables directly affects what we can learn about the investigated phenomena [23]. In the field of nonlinear spectroscopy of vibrational polaritons, an approximation is often made of the harmonic character of polariton transitions, which apparently contradicts their experimental observation, as described above [24]. If molecular transitions are treated as an ensemble of two-level systems, the concept of the excitation-induced Rabi splitting contraction can be invoked [25]. However, since nonlinear infrared spectroscopy inherently accesses molecular vibrational excitations higher than the first excited states, a theoretical description involving multi-level anharmonic emitters appears to be more suitable [26], [27], [28], [29]. The latter approach considers a ladder of polariton excitations, analogous to that used in quantum optics [30], to model the collective states of atoms and excitons strongly coupled to optical cavities. In such a situation, doubly excited polariton states 2LP, 2UP, and the combination state, LP + UP, are involved, which can be observed in the third-order nonlinear experiments either because of the mechanical anharmonicity that the vibrational polariton states acquire from their molecular components [31] or because of the differences in the magnitudes of the transition dipoles associated with the *ω*_0,LP(UP)_, *ω*_LP(UP),2LP(2UP)_, and $\omega_{\text{LP}\left(\text{UP}\right),\text{LP}+\text{UP}}$ transitions [24], as well as their relaxation rates [32] and dynamics [33].

As opposed to the quantum states represented in the position space, vibrational polaritons can be represented as states in the momentum space, i.e., as wave packets propagating across the sample. Thus, their dynamics can be qualitatively different from those of bare molecules, where the excitations are stationary. In the literature on polaritons involving semiconducting quantum dot excitons, the nonlinearity in the third- and higher-order experiments is viewed as the stimulated parametric scattering of cavity polaritons, which arises because of the weak inter-exciton interactions [30], [34], [35]. Regarding vibrational polaritons, the interaction between excitations located on different molecules is weak in solution samples; however, it can occur in densely packed polymers, which are frequently used because of their strong molecular transitions [2], [36], [37].

In the present work, we employed 2DIR spectroscopy to study third-order nonlinear excitations in vibrational polaritons. Rephasing and non-rephasing sequences of the excitation laser pulses are used to obtain the conventional single-quantum (1Q-) 2DIR spectra [38]. With these data, we can identify the excitation frequencies of the singly excited polariton states by recording the frequencies of the fundamental transitions, *ω*_0,*i*_, along the excitation frequency axis of the 1Q-2D spectrum. The so-called reverse-echo pulse sequences are used to conduct the double-quantum (2Q-) 2DIR spectroscopy [39], which accesses the second excitation manifold of the quantum system directly from the ground state, thus providing model-free information on the corresponding transition frequencies by recording the overtone transitions, *ω*_0,2*i*_. 2Q-2DIR was first introduced to investigate the intramolecular anharmonic interactions in a pair of strongly coupled C=O stretching vibrations of the transition metal carbonyl complex, where it was observed that the anharmonicity constant, Δ_*i*+*j*_, of the combination state, $\left|i+j\right\rangle$ , which is a two-particle state involving the simultaneous excitation of both vibrational modes, is appreciably larger than the anharmonicity constant of the two-particle states involving a single vibration, Δ_*i*(*j*)_ [39]. In a different example of the (detuned) vibrational modes of the amide groups in peptide molecules, Δ_*i*+*j*_ was found to be much smaller than Δ_*i*(*j*)_ because the intramolecular anharmonic interactions were rather weak compared with those of the metal carbonyl complex [40]. In these experiments, 2Q-2DIR could detect vibrational anharmonicities as small as 0.5 cm^−1^. We noted that the powerful approach of 2Q-2D spectroscopy is also extensively used in the spectroscopy of the atomic and excitonic collective excitations [30], [41], [42], [43].

## 2 Materials and methods

**Figure 1:**
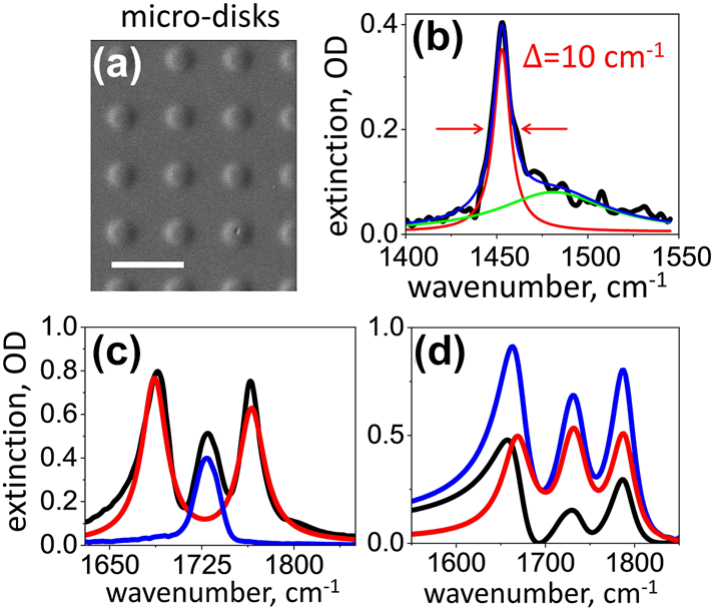
Strong coupling of PMMA vibrations to the SLR mode of a micro-disk antenna array. (a) Scanning electron micro-image of an infrared micro-disk antenna array, which serves as an open cavity. The scale bar is 5 μm. (b) Black line – the linear extinction spectrum of the array in panel a; red line – the fit to a Lorentzian profile. (c) Black line – the linear extinction spectrum of the infrared array spin-coated with 400 nm-thick PMMA film. Red line – the fit to the coupled oscillators model, blue line – the absorption spectrum of PMMA film measured off the array. (d) Electromagnetic numerical simulations of the sample. Blue line –extinction, red line – absorption, black line – scattering.

In our experiments, the carbonyl stretching (C=O) vibrations in a thin film of polymethyl methacrylate (PMMA) polymer is strongly coupled to the resonant infrared surface-lattice mode (SLR) of the periodic array of half-wavelength aluminum micro-disk antennas (see Figure 1a), which function as an open cavity [44]. Briefly, micro-disk antennas were fabricated by direct laser writing lithography (DWL 66+, Heidelberg Instruments) on a CaF_2_ substrate, as shown in Figure 1a. We used an AZ1505 photoresist and a 375 nm laser diode at 70 mW [45]. The diameter of the individual micro-disks was 1.5 μm and the height was 0.15 μm. The array had a square lattice and its periodicity was varied to tune the SLR frequency. For resonance with the C=O mode of PMMA, the period was 4.0 μm [45]. A scanning electron micro-image of the array is shown in Figure 1a.

Linear spectroscopy was performed with a Thermo-Fisher Nicollet i10 spectrometer equipped with iris diaphragms before and after the sample to limit the distribution of the incident $\vec{k}$ -vectors in the excitation light, and wire-grid polarizers were used to define the light polarization with respect to the sample [44]. The extinction spectrum measured for the normal incidence of excitation light is shown in Figure 1b. In order to match the refractive indices of the CaF_2_ substrate and superstrate, antennas were immersed in a thin liquid film of carbon tetrachloride and sandwiched with another CaF_2_ window. The bandwidth of the SLR mode obtained by fitting it to the Lorentzian profile was 10 cm^−1^, which corresponds to the quality factor *Q* ∼ 145.

To create vibrational polaritons, the micro-disk antenna arrays were spin-coated with 400 nm-thick PMMA film. Linear extinction measurements of the sample in Figure 1c showed that in addition to a relatively weak peak at the molecular transition frequency of the C=O mode, $\omega_{\text{CO}}^{\text{FTIR}}$ = 1730 cm^−1^, two strong peaks corresponding to the excitation of the LP and UP states appear in the spectrum at $\omega_{\text{LP}}^{\text{FTIR}}$ = 1688 cm^−1^ and $\omega_{\text{UP}}^{\text{FTIR}}$ = 1764 cm^−1^. The spectrum was fitted to the coupled oscillators model (the red line in Figure 1c), which confirmed that the observed polariton Rabi splitting of Ω ∼ 76 cm^−1^ corresponds to the collective coupling rate of *g* ∼ 38 cm^−1^. These numbers can be compared to the bandwidth of the molecular transitions, *γ*_PMMA_ = 23 cm^−1^, and to that of the SLR, *γ*_SLR_ = 27 cm^−1^, as obtained from fitting to the model. To conclude, our system satisfies the conditions of the strong coupling regime $g>\left(\gamma_{\text{PMMA}}+\gamma_{\text{SLR}}\right)/4$ and *g* > *γ*_PMMA_, *γ*_SLR_, as discussed in refs. [46], [47] Electromagnetic numerical simulations of the sample (FDTD, Lumerical), shown in Figure 1d, suggest that at least half of the measured extinction at the polariton transition frequencies is associated with the absorption, whereas the rest corresponds to scattering of the incident light.

**Figure 2:**
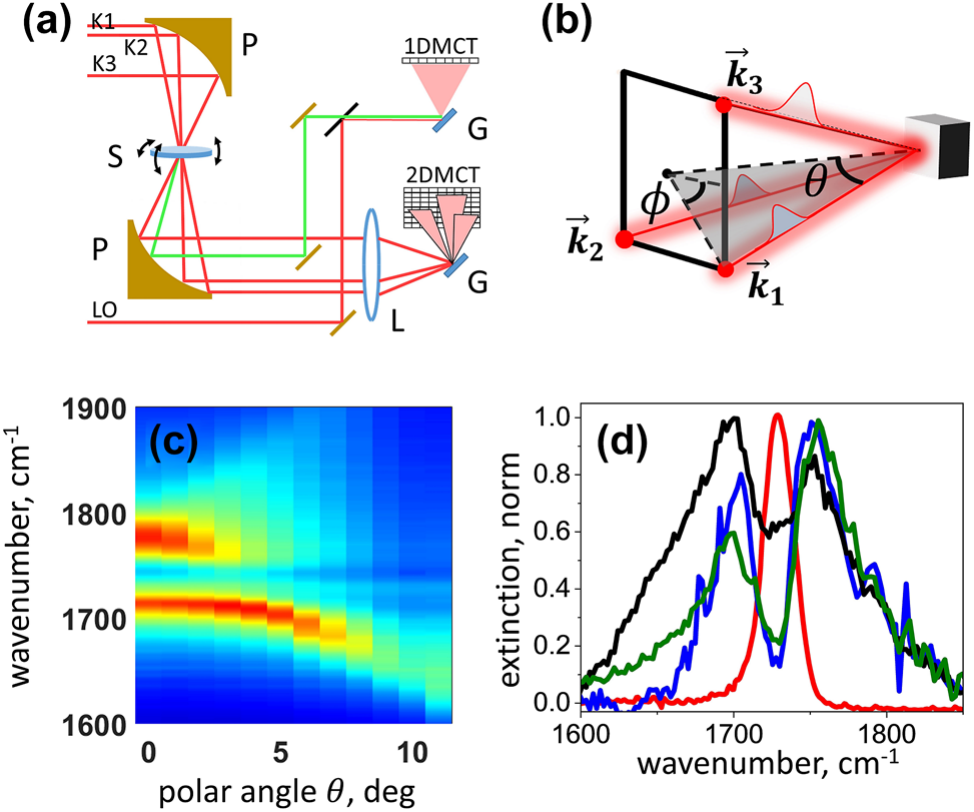
Experimental setup for 2DIR spectroscopy. (a) Schematic illustration of the 2DIR setup used to study vibrational polaritons: *P* – the parabolic mirror, *S* – sample, *L* – lens, *G* – grating, 1DMCT – the array detector used to obtain heterodyned 2DIR spectra, LO – the local oscillator, 2DMCT – the infrared camera used to obtain transmission spectra of laser pulses from each of the beams. The angles of the sample rotation used to achieve the symmetric incidence of laser pulses from all three beams with respect to the sample are indicated. (b) The incidence angles of the excitation light, characterizing the box geometry of the 2DIR setup. The laser pulses arriving at different incident angles are enumerated by their wave-vectors, ${\vec{k}}_{1-3}$ . (c) Map of the dispersion of vibrational polaritons formed by coupling between the micro-disk array open cavity tuned in resonance with CO stretching vibrations, and a thin PMMA film spin-coated on the array. (d) Normalized extinction spectrum of the three excitation laser beams ${\vec{k}}_{1-3}$ (black, blue, and olive), and bare PMMA absorption (red).

**Figure 3:**
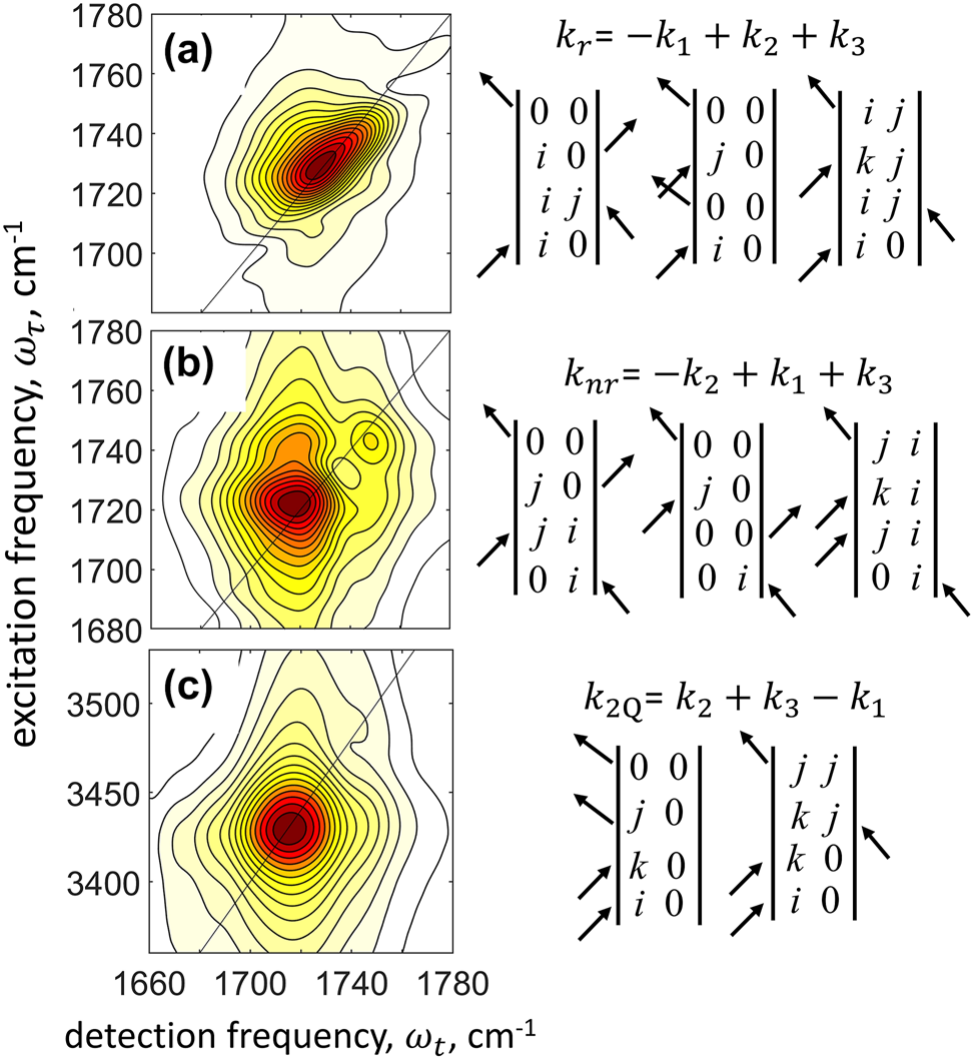
2DIR spectroscopy of PMMA. Absolute-value 1Q-2DIR rephasing (a) and non-rephasing (b) spectra, and the 2Q-2DIR spectrum (c). The corresponding time-ordered sequence of the excitation laser pulses required for fulfilling the phase-matching conditions and the associated double-sided Feynman diagrams describing the Liouville-space excitation pathways are shown on the right side of each spectrum. Here, the indices *i* and *j* represent singly excited states, whereas *k* represents doubly excited states.

The 2DIR measurements were conducted with a home-built spectrometer schematically shown in Figure 2a, which was fed by ca. 70 fs laser pulses at 4 KHz (Solstice Ace, Spectra Physics; TOPAS, NDFG, Light Conversion). Three excitation laser pulses, which are enumerated by their wave-vectors, ${\vec{k}}_{1-3}$ , arrive at the sample in a box geometry; the background-free signal emitted in the phase-matched direction is combined with the reference pulse of the local oscillator and is measured using spectral interferometry on a liquid nitrogen-cooled 64-element HgCdTe array detector (1D-MCT, Infrared Systems Development). By changing the order of the arrival of the three laser pulses in the sample, we changed the phase-matching conditions for the signal emitted in the direction of the signal collection. As such, rephasing 2DIR spectra were collected for the phase-matching conditions corresponding to the pulse ordering ${\vec{k}}_{r}$ = $-{\vec{k}}_{1}+{\vec{k}}_{2}+{\vec{k}}_{3}$ , the non-rephasing spectra for ${\vec{k}}_{nr}$ = $-{\vec{k}}_{2}+{\vec{k}}_{1}+{\vec{k}}_{3}$ , and the 2Q spectra for ${\vec{k}}_{2\text{Q}}$ = ${\vec{k}}_{2}+{\vec{k}}_{3}-{\vec{k}}_{1}$ . The corresponding double-sided Feynman diagrams, describing the Liouville-space excitation pathways [14], are shown in Figure 3. Standard approach of phasing the heterodyned 2DIR data requires a reference pump-probe measurement [14]. However, in our micro-disk samples the pump-probe data were heavily contaminated by the strong scattering of the incident light, which made such referencing formidable. Therefore, we present all our results as absolute-value 2DIR spectra, where the knowledge of the precise phase of the signal’s electric field is not required.

Since polaritons inherit the dispersive character of the SLR, their optical response strongly depends on the angle of incidence of the excitation light. In the square-box geometry of the 2DIR experiment, the direction of the incident light is characterized by the azimuthal angles *ϕ* = 45°, 135°, and 225° with respect to the principal axis of the array, as well as the polar angle *θ* with respect to the normal to the sample plane, as illustrated in Figure 2b. To understand how the angles of incidence of the excitation laser pulses used in the 2DIR experiments affect the polariton frequencies, we used FTIR spectroscopy to collect the linear spectra of the strongly coupled system with light incidence at *ϕ* = 45° and with different *θ* angles. Note that for the square lattice of the circularly symmetric disks, the incident angles *ϕ* = 45°, 135°, and 225° lead to the degenerate polariton excitations. For the dispersion measurements, the sample was mounted on a goniometer sitting on two rotation stages to allow for its precise rotation with respect to the light propagation direction within the spectrometer. The corresponding results are shown in Figure 2c. As shown in the figure, for *θ* < 5°, two well-defined transitions corresponding to the excitation of the LP and UP states are obtained. In contrast to our previous realization of the vibrational polaritons in PMMA film strongly coupled to arrays of bar-shaped gold infrared antennas [6], [36], [44], where a strong reservoir/bare-molecule transition was also observed, in the present experiments with micro-disk antennas, the SLR extinction was significantly higher and the molecular transition was not prominent.

## 3 Results

### 3.1 2DIR spectroscopy of PMMA

First, we conducted 2DIR spectroscopy of the bare PMMA polymer, which was spin-coated on the CaF_2_ glass without the micro-disk array. The results are summarized in Figure 3, where we show the three different types of the 2DIR spectra that were obtained. In the absolute-value rephasing 1Q spectrum shown in Figure 3a, the C=O stretching transition appears asymmetric, with a larger amplitude on the low-frequency side along the diagonal. Such an asymmetry was also observed earlier in the purely absorptive 1Q-2DIR spectrum of the C=O mode in PMMA [48]; however, it was not analyzed in detail. The reason for the asymmetry becomes clear after closely examining the absolute-value non-rephasing 1Q-2DIR spectrum in Figure 3b, where two separate low- and high-frequency transitions are seen at the excitation frequencies $\omega_{0,\text{CO}}^{l}$ = 1722 cm^−1^ and $\omega_{0,\text{CO}}^{h}$ = 1743 cm^−1^, respectively. These peaks arise from coupling between the transition dipoles of the closely adjacent vibrational chromophores; their frequencies and amplitudes are defined by the relative orientations and distances between these dipoles. Since in the present experiments we used a commercial PMMA resist (950 -A5 MicroChem), which can possibly involve a mixture of different isomers of the polymer strands, we did not attempt to interpret these results quantitatively in the present work. Because PMMA is known to obtain a double-helix conformation [49], the two new peaks are assigned to the split collective transitions of the *A*_1_ and *B*_2_ symmetry [50], somewhat analogously to the collective amide-I vibrational modes in helical peptides [51]. Note, however, that in peptides the corresponding transitions belong to the modes associated with the backbone, whereas in PMMA the C=O transitions reside on the polymer’s side-chains. A systematic study of the vibrational coupling between C=O transitions in PMMA will be reported elsewhere.

Despite the high-frequency C=O transition of PMMA is clearly seen in the non-rephasing 1Q-2DIR spectrum, it is almost completely buried under the shoulders of the strong low-frequency transition in the 2Q-2DIR spectrum in Figure 3c. Here, we found that the peaks appear at the excitation frequencies $\omega_{0,2\text{CO}}^{l}$ = 3429 cm^−1^ and $\omega_{0,2\text{CO}}^{h}$ = 3484 cm^−1^, such that the diagonal anharmonicities of the C=O modes are $\Delta_{\text{CO}}^{l}$ = 15 cm^−1^ and $\Delta_{\text{CO}}^{h}$ = 2 cm^−1^, respectively. The small value of $\Delta_{\text{CO}}^{h}$ is in agreement with the low amplitude of the corresponding transitions observed in the nonlinear spectra in Figure 3.

### 3.2 2DIR spectroscopy of vibro-polaritons

Next, we proceeded to perform 2DIR spectroscopy of the C=O transitions strongly coupled to the SLR mode of the micro-disk array. As discussed above, because of the polariton dispersion, it is important to ensure that all three laser pulses, ${\vec{k}}_{1-3}$ , excite the sample at the same incident angles, for which the polariton transitions are degenerate. To guarantee this condition within the box geometry of our setup, we monitored the transition frequencies of the polariton states by measuring the transmission spectrum of each laser beam with an imaging spectrograph equipped with a 128 × 128 pixel 2D-MCT detector (PhaseTech Spectroscopy, Inc.), as shown in Figure 2a. A set of the half-wavelength MgF_2_ waveplates (Karl Lambrecht Corp.) and wire-grid polarizers (Thorlabs) were used to adjust the intensity and to maintain the all-parallel polarization of the laser pulses at the sample. The sample plane with respect to the incident laser beams was precisely oriented by mounting the sample on the kinematic mount, which allows rotations in three directions, as shown in the figure. The corresponding extinction spectra obtained with each of the laser beams are shown in Figure 2d.

**Figure 4:**
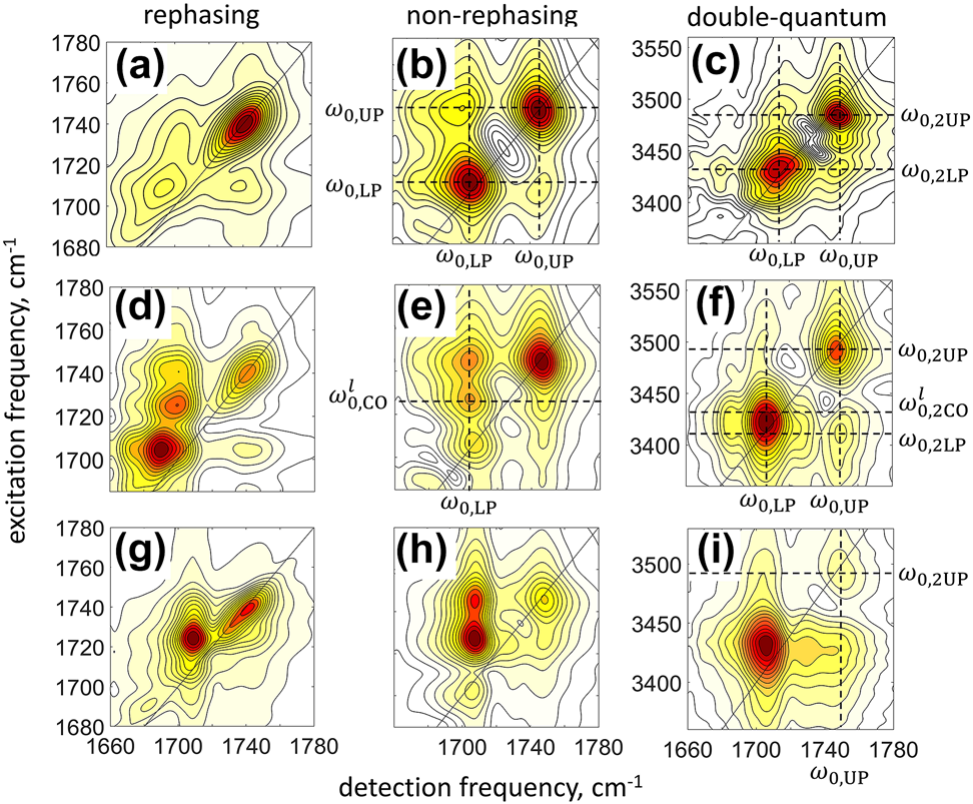
2DIR spectroscopy of vibrational polaritons. Absolute-value rephasing (left column), non-rephasing 1Q-2DIR spectra (middle column), and 2Q-2DIR spectra (right column). Three rows correspond to different detuning between the SLR and the molecular transitions achieved by the small rotation of the sample. Key transition frequencies are denoted by dashed lines.

Three sets of different 2DIR spectra of the strongly coupled system are shown in Figure 4. In the first set of the 1Q rephasing and non-rephasing spectra, shown in the top row in panels a and b, respectively, diagonal peaks associated with the polariton transitions are observed at the excitation frequencies of *ω*_0,LP_ = 1710 cm^−1^ and *ω*_0,UP_ = 1745 cm^−1^. The cross-peaks that represent photoinduced pathways corresponding to excitation of one of the polariton states and detection of another are also observed, as expected with the coupled modes. Note that in these spectra the reservoir/bare-molecule transition that is expected to appear at $\omega_{0,\text{CO}}^{l}$ = 1722 cm^−1^ is not visible. In the 2Q-2DIR spectrum in panel c, two diagonal peaks appear at the excitation frequencies of $\omega_{\tau }^{h}$ = 3488 cm^−1^ and $\omega_{\tau }^{l}$ = 3431 cm^−1^. Because the high-frequency 2Q bare-molecule C=O transition is very weak (see Figure 3c) and is not expected to interfere with the 2UP transition, we assign $\omega_{\tau }^{h}$ to *ω*_0,2UP_ and immediately obtain Δ_UP_ = 2 cm^−1^. In contrast, the low-frequency transition overlaps with the strong bare-molecule C=O transition ( $\omega_{0,2\text{CO}}^{l}$ = 3429 cm^−1^, see Figure 3c); therefore, its frequency cannot be used to reliably determine the anharmonicity of the LP. Nevertheless, similar to the 1Q spectra, in the 2Q spectrum we observe weak yet clearly visible cross-peaks between the low- and high-frequency diagonal peaks, which indicate that the latter represent excitations of the quantum states of the strongly coupled system.

A slight rotation of the sample leads to a small change in the incident angles of the laser pulses, which, in turn, results in a small change in the polariton transition frequencies; the LP is more sensitive than the UP, as expected from the dispersion relations in Figure 2c. The corresponding 2DIR data are shown in the middle row of Figure 4, where the 1Q-2DIR rephasing and non-rephasing spectra appear in panels d and e. Here, in addition to the diagonal peaks corresponding to polariton transitions at the excitation frequencies *ω*_0,LP_ = 1706 cm^−1^ and *ω*_0,UP_ = 1745 cm^−1^, and the corresponding cross-peaks between the polaritons, an additional cross-peak appears, which corresponds to the excitation of the reservoir states and the detection of the LP [36]. The weaker complementary peak, corresponding to the excitation of the reservoir and the detection of the UP, is also seen in the non-rephasing spectrum in panel e. In examining the corresponding 2Q-2DIR spectrum in Figure 4f, we found that the high- and low-frequency 2Q transitions appear at the excitation frequencies of $\omega_{\tau }^{h}$ = 3492 cm^−1^ and $\omega_{\tau }^{l}$ = 3423 cm^−1^. Similar to the data presented previously (Figure 4, top row), the high-frequency transition can be directly interpreted as *ω*_0,2UP_, leading to Δ_UP_ = −2 cm^−1^. However, also in this spectrum the low-frequency diagonal peak potentially overlaps with the bare-molecule low-frequency diagonal peak (*ω*_0,2CO_ = 3429 cm^−1^, see Figure 3c); therefore, its frequency cannot be used to reliably determine the LP anharmonicity. Interestingly, the cross-peak, which corresponds to the excitation of the low-frequency 2Q transition and the detection of the high-frequency 2Q transition, has a lower excitation frequency than that of the diagonal peak, $\omega_{\tau }^{l\to h}$ = 3412 cm^−1^. The difference in the excitation frequency between the diagonal and cross-peak is denoted in the figure by dashed lines. The cross-peak solely represents the coupled polariton states, and it is not affected by the overlapping molecular peak, as with the diagonal peak; therefore, it can be used to evaluate the anharmonicity of the LP. Here, we obtained Δ_LP_ = 0.

Further rotation of the sample with respect to the incident laser beams leads to even larger shifts of the polariton transition frequencies. In the 1Q-2DIR rephasing and non-rephasing spectra, shown in panels *g* and *h* of Figure 4, we observed that *ω*_0,LP_ = 1702 cm^−1^ and *ω*_0,UP_ = 1746 cm^−1^. We also noted that in these spectra the cross-peak corresponding to excitation of the reservoir and detection of the LP are much stronger than in the spectra shown in the top and middle rows of the figure (panels a-b and d-e, respectively). A strong diagonal peak at the bare molecule transition frequency is also observed in the corresponding 2Q-2DIR spectrum shown in panel *i*. Unlike in the previous case, here, the cross-peaks between the polaritons cannot be precisely resolved and, again, only the diagonal peak associated with the UP can be used to extract anharmonicity. We found that *ω*_0,2UP_ = 3492 cm^−1^ and, therefore, Δ_UP_ = 0.

## 4 Summary and discussion

To summarize our results, we observed that polariton modes have small to no mechanical anharmonicity; the experimental values are in the range of ca. −2 to 2 cm^−1^. Since our 2DIR spectra were obtained by Fourier transform spectroscopy, the resolution along the excitation frequency axis is given by the inverse of the scanned time interval, which in our experiments was Δ*τ* = 3.5 ps, corresponding to Δ*ω*_*τ*_ ≈ 10 cm^−1^. However, because in the spectra analyzed in the present work, we deal only with isolated transitions, their central frequency can be determined with much better precision. For example, fitting the slices of the 2DIR spectra taken along the excitation frequency axis routinely leads to fitting errors less than 1 cm^−1^. Propagating this value to estimate the error in the obtained anharmonicity values results in an error of ca. 2 cm^−1^. Our experimental results therefore place an upper bound on the polariton mechanical anharmonicity of 2 cm^−1^; however, we wish to stress again that in several spectra, no measurable mechanical anharmonicity was observed. Interestingly, unlike in the 2Q-2DIR experiments on coupled vibrational modes in bare molecules [39], [40] and in the 2Q-2DES experiments with collective atomic states [43], interacting excitons [42], and excitons strongly coupled to optical cavities [30], our 2Q-2DIR spectra do not feature the peaks associated with the combination states LP + UP, which involve simultaneous excitation of singly excited states of both LP and UP. However, we do observe cross-peaks between the doubly excited polariton states 2LP and 2UP, which are forbidden within the harmonic approximation [39].

Recently, Yuen-Zhou and co-workers derived perturbative expressions for polariton mechanical anharmonicity, which predict that Δ_UP(LP)_ scales as ∼Δ_mol_/*N*, where Δ_mol_ is the molecular anharmonicity [27], [28], [52]. Since our experimental realization of the strongly coupled system involves numerous molecules, such a predicted scaling agrees with our observations. However, the theory predicts comparable transition strengths for the doubly excited polariton states and the polariton combination state, which we interestingly did not observe. Xiang et al. [24] suggested that the nonlinear response of the molecular vibrational polaritons appears because of the differences in the dephasing rates between the singly and doubly excited polariton states, with emphasis on the polariton combination state. To test this hypothesis experimentally, fifth-order nonlinear spectroscopy is needed to directly follow the ultrafast dynamics of the doubly excited states [53], [54], which is not available with our current experimental setup.
